# Antibiotic resistance and virulence profiles of *Proteus mirabilis* isolated from broiler chickens at abattoir in South Africa

**DOI:** 10.1002/vms3.1371

**Published:** 2024-02-15

**Authors:** Tsepo Ramatla, Taole Ramaili, Kgaugelo Lekota, Kealeboga Mileng, Rendani Ndou, Malekoba Mphuthi, Ntelekwane Khasapane, Michelo Syakalima, Oriel Thekisoe

**Affiliations:** ^1^ Unit for Environmental Sciences and Management North‐West University Potchefstroom South Africa; ^2^ Gastrointestinal Research Unit Department of Surgery School of Clinical Medicine University of the Free State Bloemfontein South Africa; ^3^ Department of Animal Health, School of Agriculture North‐West University Mmabatho South Africa; ^4^ Department of Life Sciences Centre for Applied Food Safety and Biotechnology Central University of Technology Bloemfontein South Africa; ^5^ Department of Disease Control School of Veterinary Medicine University of Zambia Lusaka Zambia

**Keywords:** antimicrobial resistance, broiler chickens, *P. mirabilis*, South Africa, virulence gene

## Abstract

**Background:**

*Proteus mirabilis* has been identified as an important zoonotic pathogen, causing several illnesses such as diarrhoea, keratitis and urinary tract infections.

**Objective:**

This study assessed the prevalence of *P. mirabilis* in broiler chickens, its antibiotic resistance (AR) patterns, ESBL‐producing *P. mirabilis* and the presence of virulence genes.

**Methods:**

A total of 26 isolates were confirmed as *P. mirabilis* from 480 pooled broiler chicken faecal samples by polymerase chain reaction (PCR). The disk diffusion method was used to evaluate the antibacterial susceptibility test, while nine virulence genes and 26 AR genes were also screened by PCR.

**Results:**

All 26 *P. mirabilis* isolates harboured the *ireA* (siderophore receptors), *ptA*, and *zapA* (proteases), *ucaA, pmfA, atfA*, and *mrpA* (fimbriae), *hlyA* and *hpmA* (haemolysins) virulence genes. The *P. mirabilis* isolates were resistant to ciprofloxacin (62%) and levofloxacin (54%), while 8 (30.7%) of the isolates were classified as multidrug resistant (MDR). PCR analysis identified the *bla_CTX‐M_* gene (62%), *bla_TEM_* (58%) and *bla_CTX‐M‐2_* (38%). Further screening for AMR genes identified *mcr‐1*, *cat1*, *cat2*, *qnrA, qnrD* and *mecA*, 12%, 19%, 12%, 54%, 27% and 8%, respectively for *P. mirabilis* isolates. The prevalence of the integron integrase *intI*1 and *intI*2 genes was 43% and 4%, respectively.

**Conclusions:**

The rise of ciprofloxacin and levofloxacin resistance, as well as MDR strains, is a public health threat that points to a challenge in the treatment of infections caused by these zoonotic bacteria. Furthermore, because ESBL‐producing *P. mirabilis* has the potential to spread to humans, the presence of *bla_CTX_*_‐M_‐producing *P. mirabilis* in broilers should be kept under control. This is the first study undertaken to isolate *P. mirabilis* from chicken faecal samples and investigate its antibiotic resistance status as well as virulence profiles in South Africa.

## Graphical abstract

A total of 26 isolates were confirmed as *P. mirabilis* by 16S rRNA gene sequencing. Seven out of nine virulence genes (*ptA*, *zapA*, *ucaA*, *pmfA*, *atfA*, *mrpA* and *hlyA*) were detected in *P. mirabilis* isolates. A total of eight (30.7%) *P. mirabilis* isolates were resistant to three or more antibiotic classes. Out of 26 *P. mirabilis* isolates, 8 (30.8%) were phenotypically classified as ESBL‐producing, while 22 (84%) were classified genotypically as ESBL positive.

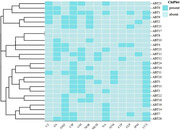

## 1 INTRODUCTION

The genus *Proteus* consists of Gram‐negative, facultatively anaerobic, heterotrophic and proteolytic bacteria that cause opportunistic human infections (Drzewiecka, 2016; Hamilton et al., 2018). *Proteus mirabilis* and *P. vulgaris* are the two main species of this genus, which were characterised by Hauser in the 19th century (Drzewiecka, 2016; Manos & Belas, 2006). Most *Proteus* spp. infections are caused by *P. mirabilis*, including catheter‐associated urinary tract infections (CAUTIs) (Wasfi et al., 2020), wound and respiratory infections (Mobley & Belas, 1995; Tsai et al., 2014; Warren et al., 1982). The *P. mirabilis* is a significant zoonotic conditional pathogen that frequently infects both humans and animals (Li et al., 2022).

The pathogenicity of *P. mirabilis* is associated with the ability to manifest virulence factors, such as the siderophore receptor *ireA* gene, haemolysing coding genes *hpm*A and *hly*A, fimbriae coding genes *atf*A, *pmf*A, *mrp*A, *uca*A, as well as the protease coding genes *zap*A and *pt*A (Li et al., 2016; Sanches et al., 2020). Previous studies reported 76% (38/50) of multidrug‐resistant (MDR) *Proteus* species isolated from broilers in China (Li et al., 2022), while in Egypt a total of 22.8% (8/35) MDR *Proteus* species isolated from healthy and diseased ducks (Algammal et al., 2021). Polymerase chain reaction (PCR) method provides a quick, accurate, and reliable epidemiological screening tool for virulence and antibiotic resistance genes in bacterial pathogens (Algammal et al., 2021).

Beta‐lactam antibiotics are a class of broad‐spectrum antibiotics consisting of all antibiotic agents that contain a beta‐lactam ring in their molecular structures and are frequently prescribed antimicrobial agents all over the world in the treatment of infections caused by Gram‐positive and Gram‐negative bacteria (Bradford, 2001; Yarima et al., 2020). Pathogenic Gram‐negative bacteria frequently produce Extended‐Spectrum Beta‐Lactamases (ESBLs), a genetic enzymatic component that contributes to their resistance to beta‐lactam antimicrobial medicines (Pitout et al., 2005; Rao et al., 2014). ESBLs are plasmid‐encoded enzymes that cause resistance to extended‐spectrum cephalosporins, monobactams and aztreonams (CDC, 2019). Recently, an increase in ESBL‐producing Enterobacteriaceae has been observed globally (Ushie et al., 2020).

*Proteus mirabilis* lacks any chromosomally encoded beta‐lactamase, resulting in complete resistance to all lactams for the wild‐type phenotype (Girlich et al., 2020). The *P. mirabilis* isolates have been described with multiple acquired resistance genes encoding narrow‐spectrum beta‐lactamases *bla_TEM_, bla_SHV_, bla_CARB_*, *bla_IRT_* (De Champs et al., 2000; Girlich et al., 2020; Naas et al., 2003). The two most dominant genes are *bla_TEM_* and *bla_CTX‐M_* (Paterson, 2006; Rajivgandhi et al., 2018). The prevalence of ESBLs of the *bla_CTX‐M_* type among *Proteus* spp. is turning into a significant threat to world health (Karapavlidou et al., 2005; Rajivgandhi et al., 2018). It has exhibited strong hydrolysis activity against cefotaxime (Auer et al., 2010).

Several *bla_OXA_* carbapenemases originating from *Acinetobacter* spp. are now emerging from *P. mirabilis* isolates, including *bla_OXA‐23_* and *bla_OXA‐58_* (Girlich et al., 2020).

The *ampC* beta‐lactamases have been found on the chromosomes of some Enterobacteriaceae (Bush et al., 1995). These cephalosporinases induce resistance to penicillins, cephalosporins, cefazolins, cefoxitins, combination *β‐*lactamases and beta‐lactamases (Jacoby, 2009).

The *P. mirabilis* isolates have been described as having multiple acquired resistance genes encoding narrow‐spectrum beta‐lactamases and quinolones (Naas et al., 2003; De Champs et al., 2000; Girlich et al., 2020; Sanches et al., 2020). Hence, various genes encoding antibiotic resistance, including beta‐lactamases and quinolones, should be screened, particularly for underreported bacteria such as *P. mirabilis*. This study investigated the occurrence of *P. mirabilis* from faecal samples of broiler chickens collected in Mafikeng City, North‐West Province, South Africa. Furthermore, this study documented the prevalence of *P. mirabilis* virulence genes as well as its phenotypic and genotypic antibiotic resistance patterns.

## 2 MATERIALS AND METHODS

### 2.1 Sample collection

Following evisceration, 2400 chicken faeces samples were randomly collected from the caeca from 2016 to 2018 in four different abattoirs around Mafikeng City in the Ngaka Modiri Molema district of North‐West Province, which is located between 25° and 28° south of the equator and 22° to 28° longitude east of the Greenwich Meridian (Ramatla et al., 2022). The faecal samples were pooled into a total of 480 samples, representing five separate broiler farms (Mileng et al., 2021).

### 2.2 Bacterial isolation and identification

For culture isolation of *Proteus mirabilis* from faeces 5 g of faecal sample was mixed with 10 mL of buffered peptone water (BPW Oxoid, Biolab, South Africa), homogenised for 2 min by vortexing and incubated for 18 to 24 h at 37°C. Loopful of liquid culture broth was streaked on Xylose lysine deoxycholate agar (XLD), incubated for 18 to 24 h at 37°C. Three to four *Salmonella*‐like colonies (red with or without black centre) suspected colonies were further streaked and purified on nutrient agar (NA). Pure colonies were then tested for their specific biochemical characteristics, and putative *P. mirabilis* isolates were selected and confirmed by 16S rRNA gene PCR.

### 2.3 DNA extraction, PCR and sequencing

The bacterial genomic DNA was extracted using the Zymo Fungal/Bacterial DNA kit following the manufacturer's instructions (Zymo Research Corp., CA, USA). A NanoDrop spectrophotometer was used to measure the DNA concentration. The bacterial universal primers (27F: AGAGTTTGATCMTGGCTCAG and 1492R: GGTTACCTTGTTACGACTT) targeting the 16S *rRNA* gene segment were used for molecular identification of the bacteria using conventional PCR. This included a total reaction volume of 25 μL, of which 12.5 μL was a 2X DreamTaq Green Master Mix (Thermo Fisher Scientific, South Africa), including 0.4 mM of each deoxynucleotide triphosphates (dATP, dCTP, dGTP and dTTP), 4 mM MgCl_2_, 2.0 μL of the template DNA, 10 μM of each oligonucleotide primer, 8.5 μL of nuclease‐free water. The ProFlex PCR System was used to carry out the PCR reactions (Applied Biosystems, USA). Amplified PCR products were electrophoresed on a 1.5% (w/v) agarose gel stained with ethidium bromide and visualised under ultraviolet (UV) light.

The 16S rRNA PCR products were sequenced at Inqaba Biotechnical Industries (Pty) Ltd., Pretoria, South Africa. FintchTV (Harb et al., 2019) was used to edit the base calls of the sequence chromatograms. Sequence identity was evaluated using the nucleotide Basic Local Alignment Search Tool (BLASTn) on the NCBI website (https://blast.ncbi.nlm.nih.gov/Blast.cg). Generated sequences were submitted to the GenBank database and were assigned accession numbers.

### 2.4 Antimicrobial susceptibility test

The phenotypic antibiotic resistance test was performed using the Kirby‐Bauer (Disc diffusion) method to assess the antimicrobial susceptibility of *P. mirabilis* isolates. Antibiotic discs cefazolin (CZ) 30 μg, amikacin (AN) 30 μg, amoxicillin‐clavulanic acid (AMC) 20/10 μg, ciprofloxacin (CIP) 5 μg, gentamicin (GM) 10 μg, norfloxacin (NOR) 10 μg, meropenem (MEM) 10 μg, nalidixic acid (NA) 30 μg, aztreonam (ATM) 30 μg, ertapenem (ETP) 10 μg, cefepime (FEP) 30 μg, imipenem (IPM) 10 μg, colistin sulphate (CS) 10 μg and levofloxacin (LVX) 5 μg (Thermofischer, South Africa) were used in this study. Isolates that display resistance to three or more antimicrobial classes were deemed as multidrug resistant as described by Ramatla et al. (2019).

Aliquots of 100 μL of bacterial suspensions were spread‐plated on Mueller‐Hinton agar, and antibiotic discs were aseptically placed on the plates using sterile forceps. Plates were incubated for 24 h at 37°C. Thereafter, the diameters of the zones of inhibition were measured in millimetres (mm). The *P. mirabilis* isolates were categorised as resistant, intermediate and susceptible based on zone of inhibition in accordance with Clinical and Laboratory Standards Institute standards (CLSI, 2018). The *E. coli* ATCC 25922 was used as a quality control strain.

### 2.5 Phenotypic detection of ESBL‐producing *Proteus mirabilis*

Confirmed *P. mirabilis* isolates were screened for phenotypic ESBL production by disc diffusion tests using cefotaxime (CTX: 30 μg), aztreonam (ATM: 30 μg), ceftazidime (CAZ: 30 μg) and ceftriaxone (CRO: 30 μg) according to CLSI guidelines (CLSI, 2018, 2020; Fayez et al., 2023). Isolates that showed resistance to one or more of these antibiotics were confirmed as ESBL production.

### 2.6 Detection of antibiotic resistance genes and *β‐*lactamase‐encoding genes

To investigate the antibiotic resistance genes, PCR amplification was performed on all *P. mirabilis* isolates. The genes encoding for quinolone (*qnrS*, *qnrA* and *qnrD*), vancomycin (*van*A), macrolide (erythromycin) (*erm*B), chloramphenicol (*catI* and *catII*), penicillin‐binding protein 2a or PBP2a (*mecA*) and colistin (*mcr*‐1 and *mcr*‐2) and the genes encoding for β‐lactams (ampC, blaCTX‐M‐8, blaCTX‐M‐9, blaCTX‐M‐25, blaCTX‐M, blaCTX‐M‐1, blaCTX‐M‐2, blaCTX‐M‐9, blaCTX‐M‐15, blaTEM, blaSHV, blaCARB and blaOXA) were investigated. The genes encoding for *β*‐lactams (*ampC*, *bla_CTX‐M‐8_, bla_CTX‐M‐9_, bla_CTX‐M‐25_, bla_CTX‐M,_ bla_CTX‐M‐1,_ bla_CTX‐M‐2,_ bla_CTX‐M‐9_, bla_CTX‐M‐15,_ bla_TEM,_ bla_SHV,_ bla_CARB_* and *bl*a*_OXA_*). Total reaction volume was 25 μL, of which 12.5 μL was a 2X DreamTaq Green Master Mix (Thermo Fisher Scientific, South Africa) including 0.4 mM of each deoxynucleotide triphosphate (dATP, dCTP, dGTP and dTTP), 4 mM MgCl_2_, 2.0 μL of the template DNA, 10 μM of each oligonucleotide primer, 8.5 μL of nuclease‐free water. PCR amplification conditions (Supplementary Table S1) were initial denaturation for 5 min at 94°C, 35 cycles of denaturation at 94°C for 45 s, annealing temperatures are shown in Supplementary Table S2, and extension at 72°C for 60 s. The final extension was carried out at 72°C for 10 min. Amplicons were electrophoresed as described above. To allow standardisation, 1 Kb and 100 bp DNA Ladders (Sigma, D7058) were utilised as molecular markers.

### 2.7 Screening integron integrase genes by multiplex PCR assay

The presence of Int (*intI*1 and *intI*2) gene‐encoding class 1 and 2 integrons was tested in all *P. mirabilis* isolates. The PCR reaction included a total reaction of 25 μL containing 12.5 μL of a 2X DreamTaq Green Master Mix (0.4 mM dATP, 0.4 mM dCTP, 0.4 mM dGTP, 0.4 mM dTTP, 4 mM MgCl_2_ and loading buffer) (ThermoFisher Scientific, South Africa), 8.5 μL of nuclease‐free water, 2.0 μL of the template DNA and 1.0 μL of each oligonucleotide primer. PCR primers and conditions for antibiotic resistance genes and class 1 and 2 integrons are shown in Supplementary Table S1. PCR reactions were performed using the ProFlex PCR System (Applied Biosystems, USA).

### 2.8 Detection of virulence genes

The virulence gene profiles of *P. mirabilis* isolates were determined using conventional PCR. The nine virulence genes screened included *ireA* (siderophore receptors), *ptA*, and *zapA*, (proteases), *ucaA*, *pmfA*, *atfA*, and *mrpA* (fimbriae), and *hlyA* and *hpmA* (haemolysins) using published PCR primers (Supplementary Table S2). To set up PCR assays for virulent genes, a total of 25 μL reaction mixture consisted of 12.5 μL of the PCR Master Mix (AmpliTaq Gold® DNA Polymerase, 0.05 units/L, Gold buffer, 930 mM Tris/HCl pH 8.05, 100 mM KCl, 0.4 mM of each dNTP, and 5 mM MgCl_2_), 10 μM of each primer, 2 μL of template DNA and 8.5 μL nuclease‐free water. As a negative control, test DNA was substituted with 2 μL of nuclease‐free water. Amplicons were electrophoresed as described above.

### 2.9 Data analysis

Data analysis was carried out using Microsoft Excel 2016 (Microsoft Corporation, Redmond, DC, USA). The sequenced *16S rRNA* gene of the 26 isolates was aligned to nucleotide sequences available in GenBank and identified by comparing them with those available in the National Centre for Biotechnology Information database (NCBI) using BLASTn (http://www.ncbi.nlm.nih.gov/BLAST/). The heatmap plots of the virulence and antibiotic resistance profile were generated using ChipPlot (https://www.chiplot.online/#).

### 2.10 Data availability

The sequenced *16S rRNA* gene of the 26 *P. mirabilis* isolates were deposited in GenBank under accession numbers ON832665–ON832690.

## 3 RESULTS

### 3.1 Identification of *P. mirabilis*

The *P. mirabilis* strains were isolated using classical microbiological tests on XLD media, which resulted in 46 suspected *P. mirabilis* colonies. A total of 26 isolates were positive on urease media with the production of pinkish‐red colouration of the medium and *P. mirabilis* was indole‐negative. The isolates were further confirmed as *P. mirabilis* by 16S rRNA gene sequencing. The query sequence of the *P. mirabilis* isolates sequenced shared high (98% to 99%) similarity with *P. mirabilis* strains IARS‐5 (JQ863236.1), swupm3 (CP071780.1), M8 16S (OL629236.1) and DS18333‐2 (OM882457.1) available in GenBank. All gene sequences generated in this study were deposited into the GenBank with accession numbers.

### 3.2 Antimicrobial susceptibility phenotypic profile

**FIGURE 1:**
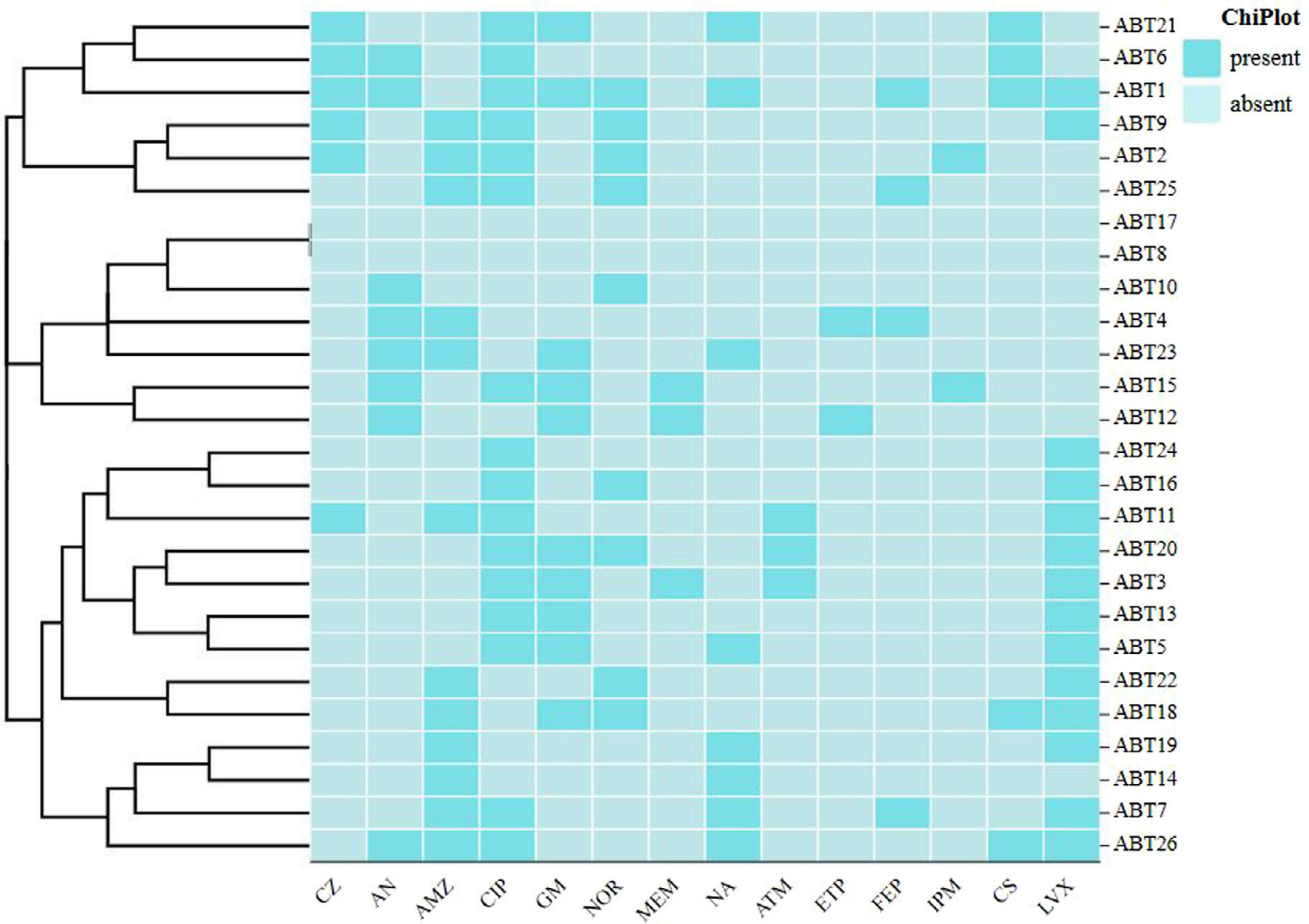
Heatmap showing the clustering of the antibiotic resistance profiles in the *P. mirabilis* isolates. Light blue and dark blue indicate absence and presence of antibiotic resistance respectively.

A total of 26 *P. mirabilis* isolates were further subjected to an antimicrobial susceptibility test to evaluate their resistance patterns (Table 1). *P. mirabilis* was found to have the highest antibiotic resistance rate against ciprofloxacin 61.5% (16/26), followed by levofloxacin 53.8% (14/26), amoxicillin‐clavulanic acid 46.2% (12/26), gentamicin 38.5% (10/26), norfloxacin 34.6% (9/26), nalidixic acid 30.8% (8/26), amikacin 30.8% (8/26), cefazolin 23.1% (6/26), colistin sulphate 19.2% (5/26), cefepime 15.4% (4/26), meropenem 11.5% (3/26), aztreonam 11.5% (3/26), ertapenem 7.7% (2/26) and imipenem 7.7% (2/26). Two *P. mirabilis* isolates (ON832672 and ON832681) were susceptible to all 14 antibiotics tested in this study. The distribution of antibiotic resistance on each *P. mirabilis* isolate is shown on the heatmap (Figure 1).

**TABLE 1:** Phenotypic antimicrobial susceptibility profile of *P. mirabilis* isolated from broiler chickens.

Group of antibiotics	Antibiotics (μg)	Abbreviation	Susceptible (%)	Intermediate (%)	Resistant (%)
Cephalosporins	Cefazolin (30 μg)	CZ	11 (42.3)	9 (34.6)	6 (23.1)
Aminoglycosides	Amikacin (30 μg)	AN	14 (53.8)	4 (15.4)	8 (30.8)
	Amoxicillin‐clavulanic acid (20/10 μg)	AMZ	11 (42.3)	1 (3.8)	12 (46.2)
	Gentamicin (10 μg)	GM	16 (61.5)	0 (0)	10 (34.6)
Fluoroquinolones	Levofloxacin (5 μg)	LVX	12 (46.2)	0 (0)	14 (53.8)
	Nalidixic acid (30 μg)	NA	17 (65.4)	1 (3.8)	8 (30.8)
	Ciprofloxacin (5 μg)	CIP	8 (30.8)	2 (7.7)	16 (61.5)
	Norfloxacin (10 μg)	NOR	11 (42.3)	6 (23.1)	9 (34.6)
Carbapenem	Meropenem (10 μg)	ME	23 (88.5)	0 (0)	3 (11.5)
	Imipenem (10 μg)	IPM	21 (80.8)	3 (11.5)	2 (7.7)
Monobactams	Aztreonam (30 μg)	ATM	19 (73.1)	4 (15.4)	3 (11.5)
	Ertapenem (10 μg)	ETP	24 (92.3)	0 (0)	2 (7.7)
4th‐generation cephalosporin	Cefepime (30 μg)	FEP	19 (73.1)	3 (11.5)	4 (15.4)
Polymyxin	Colistin sulphate (10 μg)	CS	17 (65.4)	4 (15.4)	5 (19.2)

### 3.3 Multidrug‐resistant *P. mirabilis*

A total of 8 (30.7%) *P. mirabilis* isolates were resistant to three or more antibiotic classes, which is an indication of a multidrug resistance pattern (Lv et al., 2022). Four (ABT 1, ABT 6, ABT 18 and ABT 26) isolates were resistant to four classes of antibiotics, followed by four (ABT 3, ABT 15, ABT 20, and ABT 21) isolates displaying resistance to three classes of antibiotics.

### 3.4 Antibiotic resistance genes detected in *P. mirabilis* isolates

Six out of 11 antimicrobial resistance genes were detected in the 26 *P. mirabilis* isolates. It was found that 3 (12%) out of 26 *P. mirabilis* isolates were positive for colistin resistance gene *mcr*‐1. The prevalence of *cat*1, *cat*II, *qnrA*, *qnrD* and *mecA P. mirabilis* was 5 (19%), 7 (27%), 13 (50%), 7 (27%) and 2 (8%), respectively (Table 2). The presence of the quinolone resistance genes *qnrA* and *qnrD* was detected in four *P. mirabilis* isolates. Furthermore, the chloramphenicol resistance *catI* and *catII* genes were detected in one isolate (ABT 6). Resistance genes encoding for vancomycin (*van*A), erythromycin resistance methylase (*ermB*), quinolone (*qnrS*) and colistin (*mcr‐2*) were not detected in all 26 *P. mirabilis* isolates.

**TABLE 2:** Virulence genes, antibiotic resistance phenotypes, antibiotic resistance genes and integrons of *P. mirabilis*.

Isolate code	Phenotype of resistance	Resistance genes	ESBL genes	Detection of *intI1*	Detection of *intI2*	Virulence genes
ABT1	CZ, AK, AMC, CIP, GM, NOR, MEM, NA, ATM, ETP, FEP, IPM, CS, LVX	*qnrA, qnrD, mcr‐1, catI*	*bla_CTX‐M_, bla_CTX‐M‐2_, bla_TEM_, bla_OXA_, bla_CTX‐M‐8,_ ampC*.	**+**	−	*mrpA*, *ucaA*, *atfA*, *ptA*, *zapA*
ABT2	CZ, AMC, CIP, IPM, NOR	*catII*	*bla_CTX‐M‐2_, bla_TEM_, bla_CTX‐M‐8_, bla_CTX‐M‐9_*	**+**	−	*mrpA*, *pmfA*, *atfA*, *ptA*
ABT3	CIP, GM, MEM, ATM, LVX	*mecA, qnrA, catI*	*bla_CTX‐M_, bla_CTX‐M_, bla_CTX‐M‐2_, bla_TEM_, bla_CTX‐M‐25_*	**+**	−	*mrpA*, *ucaA*, *atfA*, *ptA*
ABT4	AK, AMC, ETP, FEP	*catII*	*bla_CTX‐M‐2_, bla_SHV_, bla_CTX‐M‐8_*	−	−	*mrpA*, *atfA*, *ptA*, *zapA*
ABT5	CIP, GM, NA, LVX	*qnrA, qnrD*	*bla_CTX‐M‐2_, bla_SHV_, bla_CTX‐M‐8,_ ampC*	**+**	**+**	*mrpA*, *ucaA*, *atfA*, *ptA*
ABT6	CZ, AK, CIP, CS	*qnrA, catI, catII*	*bla_CTX‐M_, bla_CTX‐M‐2_, bla_TEM_, bla_SHV_, bla_CTX‐M‐9_*	−	−	*mrpA*, *pmfA*, *atfA*, *ptA*
ABT7	AMC, CIP, NA, FEP, LVX	*qnrA*	*bla_CTX‐M_, bla_CTX‐M‐2_, bla_TEM_, bla_SHV_, bla_CTX‐M‐8_*	**+**	−	*mrpA*, *ucaA*, *atfA*, *ptA*, *zapA*
ABT8	CZ, AMC, CIP, LVX, NOR	−	*bla_CTX‐M_*, *bla_CTX‐M‐2_, bla_TEM_, blaSHV, ampC*.	−	−	*mrpA*, *ucaA*, *atfA*, *ptA*, *zapA*
ABT9	CZ, AMC, CIP, LVX, NOR	*qnrD*	*bla_CTX‐M_, bla_CTX‐M‐2_, bla_TEM_, bla_SHV_, bla_CTX‐M‐9_*	−	−	*mrpA*, *ucaA*, *atfA*, *ptA*
ABT10	AK, NOR	−	*bla_CTX‐M_, bla_SHV_, bla_CTX‐M‐8,_ ampC*	−	−	*mrpA*, *atfA*, *ptA*
ABT11	CZ, AMC, CIP, ATM, LVX	*qnrA, mcr‐1*	*bla_CTX‐M_*	**+**	−	*mrpA*, *ucaA*, *atfA*, *ptA*, *zapA*
ABT12	AK, GM, MEM, ETP	*qnrA, qnrD, mcr‐1*	−	**+**	−	*mrpA*, *atfA*, *ptA*
ABT13	CIP, GM, LVX	−	*bla_CTX‐M_, bla_CTX‐M‐2_, bla_TEM_*	**+**	−	*mrpA*, *atfA*, *ptA*
ABT14	AMC, NA	*mecA*	*bla_TEM_, bla_SHV_*	−	−	*mrpA*, *ucaA*, *atfA*, *ptA*
ABT15	AK, CIP, GM, MEM, IPM	*qnrA, qnrD, catII*	*bla_TEM_, bla_OXA_, bla_CTX‐M‐8,_ ampC*	**+**	−	*mrpA*, *atfA*, *ptA*
ABT16	CIP, LVX, NOR	*catII*	*bla_CTX‐M_, bla_TEM_, bla_CTX‐M‐9_*	−	−	*mrpA*, *atfA*, *ptA*
ABT17	−	*catII*	*bla_CTX‐M_, bla_TEM_*	−	−	*mrpA*, *atfA*, *ptA*
ABT18	AMC, GM, NOR, CS, LVX	*qnrA*	*bla_CTX‐M_, bla_CTX‐M‐9_, bla_CTX‐M‐25_*	−	−	*mrpA*, *ucaA*, *atfA*, *ptA*, *zapA*
ABT19	AMC, NA	−	*bla_CTX‐M_*	−	−	*mrpA*, *atfA*, *ptA*
ABT20	CIP, GM, NOR, ATM, LVX	*catI*	*bla_CTX‐M_*	−	−	*mrpA*, *atfA*, *ptA*, *zapA*
ABT21	CIP, GM, NA, CS	*qnrD, catI*	*bla_TEM_*	**+**	−	*mrpA*, *pmfA*, *atfA*, *ptA*, *zapA*
ABT22	AMC, LVX, NOR	*qnrA*	*bla_CTX‐M_, bla_TEM_, bla_CTX‐M‐8_, bla_CTX‐M‐9_*	−	−	*mrpA*, *ucaA*, *atfA*, *ptA*
ABT23	AK, AMC, GM, NA	*qnrA, qnrD*	*bla_CTX‐M_*,	−	−	*mrpA*, *ucaA*, *atfA*, *ptA*
ABT24	CIP, LVX	*CatII*	*bla_CTX‐M‐9_*	**+**	−	*mrpA*, *atfA*, *ptA*
ABT25	AMC, CIP, FEP, NOR	*qnrA*	*bla_CTX‐M_*,	−	−	*mrpA*, *ucaA*, *pmfA*, *atfA*, *ptA*, *zapA*
ABT26	AK, AMC, CIP, NA, CS, LVX	*qnrA*	*bla_CTX‐M‐8_*	−	−	*mrpA*, *atfA*, *ptA*, *zapA*

CZ = cefazolin, AK = amikacin, AMC = amoxicillin‐clavulanic acid, CIP = ciprofloxacin, GM = gentamicin, NOR = norfloxacin, ME = meropenem, NA = nalidixic acid, ATM = aztreonam, ETP = ertapenem, FEP = cefepime, IPM = imipenem, CS = colistin sulphate, levofloxacin LVX.

### 3.5 Prevalence of ESBL genes among ESBL‐producing *P. mirabilis* isolates

Out of 26 *P. mirabilis* isolates, 8 (30.8%) were phenotypically classified as ESBL‐producing, while 22 (84%) were classified genotypically as ESBL positive. The ESBL‐producing *P. mirabilis* isolates had the highest ESBL genes encoding for *bla_CTX‐M‐8_* 6 (23.1%), *bla_CTX‐M_* 5 (19.2%), *bla_CTX‐M‐2_* 4 (15.4%), *bla_TEM_* 4 (15.4%), *bla_CTX‐M‐9_* 3 (11.5%) and *bla_OXA_* 2 (7.7%). A total of 4 (15.4%) ESBL‐producing *P. mirabilis* isolates harboured the *amp*C gene.

### 3.6 Distribution of ESBL genotypes

The ESBL genotype distribution is presented in Table 2 for isolates producing ESBL. A total of 4 isolates (15.38%) harboured genotypic resistance pattern combinations of *bla_CTX‐M_*, *bla_CTX‐M‐2_*, *bla_TEM_* and *bla_SHV_*. The co‐existence of *bla_CTX‐M_* types was observed in 12 (46.15%) of the isolates.

### 3.7 Detection of integron integrase genes

Twenty‐six *P. mirabilis* strains were examined for the presence of two types of integrase genes. We observed the presence of the integrase gene (*intI1*) in 11 (42%) isolates, while 1 (C1HU 5) (4%) isolate harboured both *intI1* and *intI*2 genes.

### 3.8 Detection of virulence genes

**FIGURE 2:**
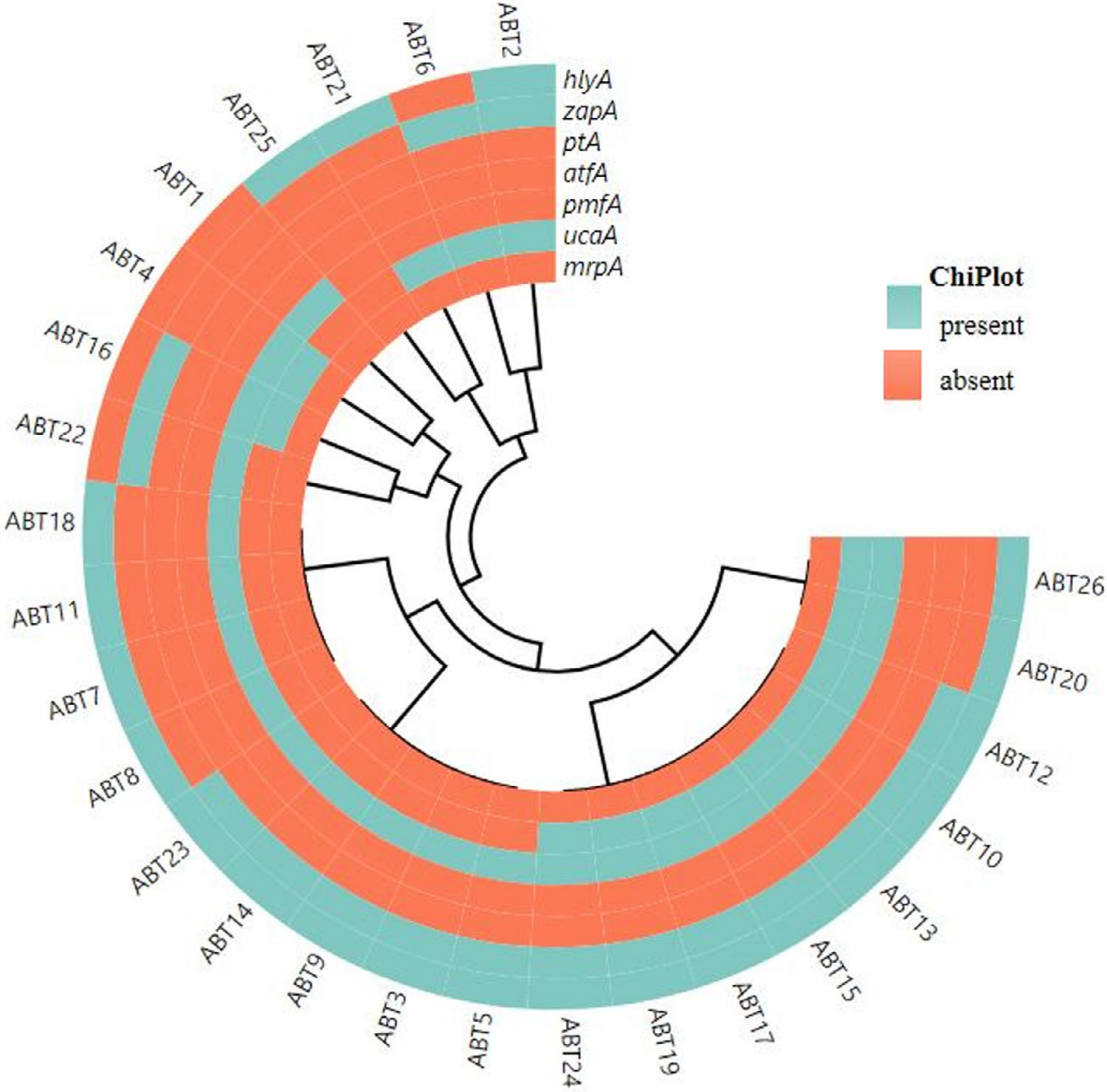
Heatmap map showing clustering of virulence genes detected in 26 *P. mirabilis* isolates. Orange and green indicates the presence and absence of virulence genes, respectively.

Seven out of nine virulence genes (*ptA*, *zapA*, *ucaA*, *pmfA*, *atfA*, *mrpA*, and *hlyA*) were detected in *P. mirabilis* isolates (*n* = 26) (Table 2). Majority of these isolates harboured virulence genes; *ptA* 26 (100%), *atfA* 26 (100%), and *mrpA* 26 (100%) (Figure 2), and virulence genes were just randomly spread to all abattoirs. Other genes detected included *zapA*, *ucaA*, *pmfA*, and *hlyA*, with prevalence of 10 (39%), 12 (46%), 4 (15%) and 5 (19%), respectively. Virulence genes such as *ireA* and *hpmA* were not detected in the *P. mirabilis* isolates.

## 4 DISCUSSION

There are several published studies reporting *P. mirabilis* infections from human samples in South Africa as well as its antibiotic resistance patterns (Fourie et al., 2021; Irusen et al., 2021; Nana et al., 2021; Pitout et al., 1998). However, there is a scarcity of studies reporting the prevalence of *P. mirabilis* in animals. Hence, the current study sought to fill in such an information gap by investigating the occurrence of *P. mirabilis* in broiler chicken samples. South Africa. The microbiological culture‐based approach and *16S* rRNA gene sequencing were used to successfully identify 26 (5.4%) of the *P. mirabilis* strains out of 480 pooled faecal samples in this study. Pitout et al. (1998) identified *P. mirabilis* as the cause of human patient wounds from hospitals in Cape Town, Durban, Soweto and Pretoria, while Botes (1964) identified these bacteria as the causative agent of an outbreak in calves. However, a number of investigations have been carried out in various nations to ascertain the incidence and presence of *P. mirabilis* in chicken samples. An overall isolation rate of 7.07% for *P. mirabilis* was recorded in Shandong Province, China (Li et al., 2022). While *P. mirabilis* was isolated from chicken droppings in commercial poultry farms in Bangladesh with a prevalence of 39% (Nahar et al., 2014), while 46% of chicken carcasses were confirmed to have *P. mirabilis* infection in Pakistan (Ishaq et al., 2022). Based on empirical evidence, *Proteus* species are linked to human diseases, animal infections and the bacterial contamination of chicken products (Nahar et al., 2014).

Animal‐derived *P. mirabilis* is a significant zoonotic pathogen that carries numerous virulence genes (Armbruster et al., 2019; Li et al., 2022). In the current study, *P. mirabilis* isolates contained the *ptA*, *zapA*, *ucaA*, *pmfA*, atfA, *mrpA* and *hlyA* genes. The *ptA*, *atfA* and *mrpA* genes were the predominant virulence genes detected in this study, with the highest detection rate of 100%. The findings of this study also demonstrated that the *ireA, hlyA* and *hpmA* genes were present in all *P. mirabilis* isolates, which is similar to the study conducted by Sanches et al. (2019), whereby all samples harboured the *ireA* and *hpmA* genes. However, our results are in contrast with observations from previous studies, where very few isolates of *P. mirabilis* harboured these genes (Cestari et al., 2013; Lazm et al., 2018; Li et al., 2022; Uphoff & Welch, 1990). Variations may result from a difference in study time, geographic properties, sample types, sample sizes or identification methods.

Previously curable bacterial infections are now frequently untreatable or necessitate the use of antibiotics as a last resort (Sherchan et al., 2015; Wu et al., 2021). The *P. mirabilis* drug resistance has become increasingly serious in recent years (Li et al., 2022). This study revealed a higher antibiotic resistance rate against ciprofloxacin (62%) and the lowest rates of 8% for each of ertapenem and imipenem in the *P. mirabilis* isolates. The *P. mirabilis* was 39% resistant to gentamicin and 31% to amikacin from the perspective of aminoglycoside resistance. Ciprofloxacin (62%) was the antibiotic in this study with the highest level of fluoroquinolone resistance. This AR prevalence against ciprofloxacin is higher when compared to the findings of other studies, whereby Kwiecińska‐Piróg et al. (2013) reported only 40% AR in Poland. Hernandez et al. (2000) indicated that 16.2% of isolates were resistant to ciprofloxacin, while Ko et al. (2008) recorded 13.6% of *P. mirabilis* AR isolates in South Korea. The availability and accessibility of antimicrobial drugs such as streptomycin, ciprofloxacin, erythromycin, tetracycline and gentamicin in open markets to treat chickens (layer/broiler) represents a barrier to lowering antimicrobial resistance in poultry farms (Hasan et al., 2011; Nahar et al., 2014) and thus poses a public health concern.

The *P. mirabilis* isolates from broiler chickens are of public health concern since infections with these bacteria can result in failure to respond to the commonly used fluoroquinolone (ciprofloxacin), which increases mortality and delays treatment outcomes (Jamil et al., 2017). Quinolone resistance is a current global issue in both human and veterinary medicine (Seo & Lee, 2019). Quinolone resistance genes mediated by the plasmids promote the spread of the multidrug resistance phenotype (Racewicz et al., 2022). The results obtained from this study revealed that one isolate was phenotypically and genotypically resistant to quinolones. The presence of the quinolone *qnr*A gene remains extremely rare in *P. mirabilis* (Girlich et al., 2020). However, in this study, quinolone‐resistant genes, *qnrA* (54%) and *qnrD* (27%), were detected in *P. mirabilis* isolates. These results are very similar to those of Mokracka et al. (2012) where they reported 27% of isolates consisting of *qnr*D in Europe. Chloramphenicol resistance genes [chloramphenicol acetyltransferase (*cat1* 19% and *catII* 12%)] were also detected in this study. Similar findings were reported by Santos et al. (2014), where they detected the *cat1* gene in 14.3% of *P. mirabilis* isolates in Brazil. One *P. mirabilis* ABT 12 strain harboured three (CIP, NA and LVX) antibiotic resistance and quinolone resistance genes (*qnrA* and *qnrD*).

The *β*‐lactamase identified in Enterobacteriaceae has also been described in *Proteus* spp. (Girlich et al., 2020). The *bla_CTX‐M‐1_*, *bla_CTX‐M‐2_*, *bla_CTX‐M‐8_*, *bla_CTX‐M‐9_* and *bla_CTX‐M‐25_* enzymes are classified into five major phylogenetic groups (Girlich et al., 2020). We report the first detection of ESBL‐*P. mirabilis* in broiler chickens in South Africa, characterised by both phenotypic and genotypic resistance. This study revealed that, of the 26 isolates of *P. mirabilis* tested using genotyping, 22 (84%) were identified as ESBL positive, while 8 (30.8%) were classified phenotypically as ESBL‐producing. The genotypic method employing specific PCR amplification of resistance genes appears to be 100% sensitive and specific. There is a significant difference between phenotypic and genotypic methods, which emphasises the importance of molecular methods in interpreting antimicrobial resistance profiles. Detection of resistance differs by phenotypic method due to its lower sensitivity and environmental factors on resistance incidence (Somily et al., 2015; Tewari et al., 2019).

The *bla_CTX_* gene was the highest detected gene (62% of ESBL‐producing *P. mirabilis* isolates), followed by the *bla_TEM_* gene (58%) and the *bla_CTX‐M‐2_* gene (38%). This is lower than the results reported in other countries, where 52% (147/282) and 12.86 % (75/583) ESBL genes were detected in *P. mirabilis* from chicken, pork and beef in Italy (Pagani et al., 2002) and in chicken, pork, beef and UTI‐CA in Brazil, respectively (Sanches et al., 2023). Among our isolates, *bla_CTX‐M_, bla_TEM_* and *bla_CTX‐M‐2_* were the most commonly detected ESBL genotypes. This agrees with other studies conducted in Korea, Italy and Brazil that reported the presence of genes from *P. mirabilis* (Ahn et al., 2017; Luzzaro et al., 2001; Sanches et al., 2023) but differs with the study conducted in Egypt (Shaaban et al., 2022), where the *bla_SHV_*, *bla_ampC_* and *bla_VIM‐1_* were the most detected genes. Multiple ESBL resistance genes may impart resistance to β‐lactamases regardless of reduced expression of one or more of those genes (Gundran et al., 2019).

Most of the poultry isolates have more than one *bla_CTX‐M_* group. Since *bla_CTX‐M_* has many homologous regions, this co‐existence may result in the emergence of recombinant enzymes (Gundran et al., 2019). In total, five *P. mirabilis* (19.2%) have three types of *bla_CTX‐M_*. The bla*_CTX‐M‐9_* gene was detected in three isolates in this study. Animals have been linked to *bla_CTX‐M‐9_*‐like enzymes either directly or indirectly in a number of countries, including Brazil (Sanches et al., 2023) and Korea (Song et al., 2011). The use of antimicrobials on farms is the major cause of the appearance of ESBL (CDC, 2019; Monyama et al., 2023; Wang et al., 2023). Our findings highlight a higher proportion of *bla_CTX‐M_*‐positive *P. mirabilis* isolates, further illuminating the propagation of this resistance gene among environmental *P. mirabilis* isolates.

It has been discovered that *P. mirabilis* is prone to spreading plasmid‐mediated ESBLs, including *bla_TEM_*‐type derivatives that are active against expanded‐spectrum cephalosporins (Biendo et al., 2005; Bonnet et al., 1999). In the present study, 58% *bla_TEM_*‐positive *P. mirabilis* were detected by PCR. *Proteus mirabilis* has been described as harbouring a large number of *bla_TEM_* variants, including *bla_TEM‐1_* and *bla_TEM‐2_* penicillinases (Pagani et al., 2003).

The ESBLs and *ampC* beta‐lactamases are produced by members of the Enterobacteriaceae in food‐producing animals (von Tippelskirch et al., 2018). In this study, four isolates harboured the *ampC* gene. These results are different from the study conducted by Lin et al. (2019) in Northern Taiwan, whereby all isolates tested negative for the *ampC* gene. In the study conducted in Egypt, the prevalence of the *ampC* gene in *P. mira*bilis strains was 28.3% from the samples obtained from the hospital (Fam et al., 2013). In another study conducted in Egypt, one *P. mirabilis* isolate harboured the *ampA* gene (Helmy & Wasfi, 2014). *Proteus mirabilis* lacks a chromosomal *bla_ampC_* gene (Helmy & Wasfi, 2014; Jacoby, 2009). Though, it has been found that *P. mirabilis* contains carbapenemases (Ohno et al., 2017). In this study, carbapenemase genes were detected in two (7.6%) *P. mirabilis* isolates. Since direct transmission of extended‐spectrum cephalosporin‐resistant isolates from animals to humans has been documented, this is a serious concern for veterinary medicine as well as human medicine (Marshall & Levy, 2011; Ramatla et al., 2024).

Since MDR bacterial infections are associated with high death rates, emerging antibiotic resistance is currently recognised as one of the most critical public health issues. The *P. mirabilis* isolates harboured a high number of antibiotic‐resistant strains in our study, with the highest proportion of drug resistance to 4 out of 4 classes of antibiotics. A total of 8 (30.7%) isolates were resistant to ≥3 antibiotic classes, and four isolates were resistant to 4 classes of antibiotics. The MDR prevalence detected in the current study is lower compared to previously reported prevalence of 46% and 78.13% for *P. mirabilis* isolates in China (Li et al., 2022) and Brazil (Sanches et al., 2019), respectively. However, the results in this study were higher than the reported prevalence of 28.7% antibiotic‐resistant isolates in Northeast China (Sun et al., 2020). The MDR‐associated *intI1* gene was found in 35% of the isolates in the current study. One *P. mirabilis* isolate harboured the *intI2* gene with four (CIP, GM, NA and LVX) antibiotic‐resistant and quinolone‐resistant genes (*qnrA* and *qnrD*). The prevalence of the integron integrase *intI1* and *intI2* genes was 42% and 4%, respectively. A study conducted by Mirzaei et al. (2021) showed a high prevalence of 60% for *intI1* and 25% for *intI2* genes compared to the results obtained in the current study. The high prevalence of MDR *P. mirabilis* isolates detected in this study raises real concern about substantial health risks since these strains may have a chance of contaminating food products and then spreading to humans. Future studies should seek to investigate the relationship between *P. mirabilis* isolates from animals and humans in order to understand the origin of antibiotic resistance development, as most antibiotics are used to treat infections in humans, while in animals such as chickens, they are used as growth promoters.

## 5 CONCLUSION

Human infections of *P. mirabilis* have already been reported by several studies in South Africa, demonstrating its public health and ‘One Health’ importance due to the development of antibiotic resistance (Fourie et al., 2021; Irusen et al., 2021; Nana et al., 2021; Pitout et al., 1998). In this study, *P. mirabilis* was isolated from broiler chicken faecal samples. We further confirmed that these *P. mirabilis* isolates possess virulence genes suggestive of a potential threat to food safety in chicken products. The findings of this study also paint a clear picture of widespread antibiotic resistance to ciprofloxacin, amoxicillin‐clavulanic acid and gentamicin, which potentially poses a serious threat to public health. This study also detected three ESBL genes, bla*_CTX‐M‐8_*, bla*_CTX‐M_*, bla*_CTX‐M‐2_*, bla*_TEM_*, bla*_OXA_* 2 and bla*_CTX‐M‐9_*, in *P. mirabilis* isolates. This is the first study to detect *P. mirabilis* in chicken faeces in South Africa. Data generated in this study fills in the information gap about the zoonotic and ‘One Health’ significance of this bacterium in South Africa. This highlights the importance of formulation of a consolidated ‘One Health’ strategy to manage *P. mirabilis* infections by both the human and animal health sectors.

## AUTHOR CONTRIBUTIONS

Tsepo Ramatla: conceptualisation, data curation, investigation, methodology, resources, visualisation and writing original draft. Taole Ramaili, Kealeboga Mileng and Ntelekwane Khasapane: conceptualisation, data curation, investigation, methodology, resources and visualisation. Kgaugelo Lekota: conceptualisation, data curation, methodology and writing review & editing. Rendani Ndou and Malekoba Mphuthi: validation, formal analysis and writing review & editing. Oriel Thekisoe and Michelo Syakalima: validation and writing review & editing. All authors have read and agreed to the published version of the manuscript.

## CONFLICT OF INTEREST STATEMENT

The authors declare that there is no conflict of interest among them.

## FUNDING

No funding was received for this study.

### PEER REVIEW

The peer review history for this article is available at https://www.webofscience.com/api/gateway/wos/peer-review/10.1002/vms3.1371.

## ETHICS STATEMENT

The animal and human experimentation and animal care procedures ethical committee of NWU approved the study (Ethics number: NWU‐00511‐18‐A5).

## Supporting information

SUPPORTING INFORMATION

## Data Availability

Data available on request from the authors

## References

[vms31371-bib-0001] Ahn, J. Y. , Ann, H. W. , Jeon, Y. , Ahn, M. Y. , Oh, D. H. , Kim, Y. C. , & Kim, J. M. (2017). The impact of production of extended‐spectrum *β‐*lactamases on the 28‐day mortality rate of patients with *Proteus mirabilis* bacteremia in Korea. BMC Infectious Diseases, 17(1), 1–10. 28468622 10.1186/s12879-017-2431-8 PMC5415711

[vms31371-bib-0002] Algammal, A. M. , Hashem, H. R. , Alfifi, K. J. , Hetta, H. F. , Sheraba, N. S. , Ramadan, H. , & El‐Tarabili, R. M. (2021). *atp D* gene sequencing, multidrug resistance traits, virulence‐determinants, and antimicrobial resistance genes of emerging XDR and MDR‐*Proteus mirabilis* . Scientific Reports, 11(1), 9476. 33947875 10.1038/s41598-021-88861-w PMC8096940

[vms31371-bib-0003] Armbruster, C. E. , Forsyth, V. S. , Johnson, A. O. , Smith, S. N. , White, A. N. , Brauer, A. L. , & Mobley, H. L. (2019). Twin arginine translocation, ammonia incorporation, and polyamine biosynthesis are crucial for *Proteus mirabilis* fitness during bloodstream infection. PLoS Pathogens, 15(4), e1007653. 31009518 10.1371/journal.ppat.1007653 PMC6497324

[vms31371-bib-0004] Auer, S. , Wojna, A. , & Hell, M. (2010). Oral treatment options for ambulatory patients with urinary tract infections caused by extended‐spectrum‐*β*‐lactamase‐producing *Escherichia coli* . Antimicrobial Agents and Chemotherapy, 54(9), 4006–4008. 20585127 10.1128/AAC.01760-09 PMC2935012

[vms31371-bib-0005] Biendo, M. , Thomas, D. , Laurans, G. , Hamdad‐Daoudi, F. , Canarelli, B. , Rousseau, F. , Castelain, S. , & Eb, F. (2005). Molecular diversity of *Proteus mirabilis* isolates producing extended‐spectrum β‐lactamases in a French university hospital. Clinical Microbiology and Infection, 11(5), 395–401. 15819867 10.1111/j.1469-0691.2005.01147.x

[vms31371-bib-0006] Bonnet, R. , De Champs, C. , Sirot, D. , Chanal, C. , Labia, R. , & Sirot, J. (1999). Diversity of TEM mutants in *Proteus mirabilis* . Antimicrobial Agents and Chemotherapy, 43(11), 2671–2677. 10543745 10.1128/aac.43.11.2671 PMC89541

[vms31371-bib-0007] Botes, H. J. W. (1964). *Proteus Mirabilis* as a cause of disease in calves. Journal of the South African Veterinary Association, 35(2), 187–192.

[vms31371-bib-0008] Bradford, P. A. (2001). Extended‐spectrum *β*‐lactamases in the 21st century: Characterization, epidemiology, and detection of this important resistance threat. Clinical Microbiology Reviews, 14(4), 933–951. 11585791 10.1128/CMR.14.4.933-951.2001 PMC89009

[vms31371-bib-0009] Bush, K. , Jacoby, G. A. , & Medeiros, A. A. (1995). A functional classification scheme for beta‐lactamases and its correlation with molecular structure. Antimicrobial Agents and Chemotherapy, 39(6), 1211–1233. 7574506 10.1128/aac.39.6.1211 PMC162717

[vms31371-bib-0010] CDC . (2019). ESBL‐producing Enterobacterales in Healthcare Settings [https://www.cdc.gov/hai/organisms/ESBL.html]

[vms31371-bib-0011] Cestari, S. E. , Ludovico, M. S. , Martins, F. H. , da Rocha, S. P. D. , Elias, W. P. , & Pelayo, J. S. (2013). Molecular detection of HpmA and HlyA hemolysin of uropathogenic *Proteus mirabilis* . Current Microbiology, 67, 703–707. 23884594 10.1007/s00284-013-0423-5

[vms31371-bib-0012] Clinical and Laboratory Standards Institute (CLSI) . (2018). Performance standards for antimicrobial susceptibility testing (28th ed.). CLSI supplement M100. Clinical and Laboratory Standards Institution.

[vms31371-bib-0013] Clinical and Laboratory Standards Institute (CLSI) . (2020). Performance standard antimicrobial testing (30th ed.). CLI supplement M100. Clinical and Laboratory Standards Institution.

[vms31371-bib-0014] De Champs, C. , Bonnet, R. , Sirot, D. , Chanal, C. , & Sirot, J. (2000). Clinical relevance of *Proteus mirabilis* in hospital patients: A two‐year survey. Journal of Antimicrobial Chemotherapy, 45(4), 537–539. 10747835 10.1093/jac/45.4.537

[vms31371-bib-0015] Drzewiecka, D. (2016). Significance and roles of *Proteus* spp. bacteria in natural environments. Microbial Ecology, 72(4), 741–758. 26748500 10.1007/s00248-015-0720-6 PMC5080321

[vms31371-bib-0016] Fam, N. , Gamal, D. , El Said, M. , El Defrawy, I. , El Dadei, E. , El Attar, S. , Sorur, A. , Ahmed, S. , & Klena, J. (2013). Prevalence of plasmid‐mediated *ampC* genes in clinical isolates of Enterobacteriaceae from Cairo, Egypt. British Microbiology Research Journal, 3(4), 525–537.

[vms31371-bib-0017] Fayez, M. , Elmoslemany, A. , Al Romaihi, A. A. , Azzawi, A. Y. , Almubarak, A. , & Elsohaby, I. (2023). Prevalence and risk factors associated with multidrug resistance and extended‐spectrum β‐lactamase producing *E. coli* isolated from healthy and diseased cats. Antibiotics, 12(2), 229. 36830140 10.3390/antibiotics12020229 PMC9951988

[vms31371-bib-0018] Fourie, J. L. , Claassen, F. M. , & Myburg, J. J. (2021). Causative pathogens and antibiotic resistance in community acquired urinary tract infections in central South Africa. South African Medical Journal, 111(2), 124–128. 33944722 10.7196/SAMJ.2021.v111i2.14905

[vms31371-bib-0019] Girlich, D. , Bonnin, R. A. , Dortet, L. , & Naas, T. (2020). Genetics of acquired antibiotic resistance genes in *Proteus* spp. Frontiers in Microbiology, 11, 256. 32153540 10.3389/fmicb.2020.00256 PMC7046756

[vms31371-bib-0020] Gundran, R. S. , Cardenio, P. A. , Villanueva, M. A. , Sison, F. B. , Benigno, C. C. , Kreausukon, K. , Pichpol, D. , & Punyapornwithaya, V. (2019). Prevalence and distribution of *bla CTX‐M, bla SHV, bla TEM* genes in extended‐spectrum β‐lactamase‐producing *E. coli* isolates from broiler farms in the Philippines. BMC Veterinary Research, 15, 1–8. 31277658 10.1186/s12917-019-1975-9 PMC6612079

[vms31371-bib-0021] Hamilton, A. L. , Kamm, M. A. , Ng, S. C. , & Morrison, M. (2018). *Proteus* spp. as putative gastrointestinal pathogens. Clinical Microbiology Reviews, 31(3), e00085–e00117. 29899011 10.1128/CMR.00085-17 PMC6056842

[vms31371-bib-0022] Harb, A. , Abraham, S. , Rusdi, B. , Laird, T. , O'dea, M. , & Habib, I. (2019). Molecular detection and epidemiological features of selected bacterial, viral, and parasitic enteropathogens in stool specimens from children with acute diarrhea in Thi‐Qar Governorate, Iraq. International Journal of Environmental Research and Public Health, 16(9), 1573. 31064051 10.3390/ijerph16091573 PMC6539995

[vms31371-bib-0023] Hasan, B. , Faruque, R. , Drobni, M. , Waldenström, J. , Sadique, A. , Ahmed, K. U. , & Alam, M. (2011). High prevalence of antibiotic resistance in pathogenic *Escherichia coli* from large‐and small‐scale poultry farms in Bangladesh. Avian Diseases, 55(4), 689–692. 22312993 10.1637/9686-021411-Reg.1

[vms31371-bib-0024] Helmy, M. M. , & Wasfi, R. (2014). Phenotypic and molecular characterization of plasmid mediated *AmpC β*‐lactamases among *Escherichia coli*, *Klebsiella* spp., and *Proteus mirabilis* isolated from urinary tract infections in Egyptian hospitals. BioMed Research International, 2014, 171548. 25003107 10.1155/2014/171548 PMC4070535

[vms31371-bib-0025] Hernandez, J. R. , Martinez‐Martinez, L. , Pascual, A. , Suárez, A. I. , & Perea, E. J. (2000). Trends in the susceptibilities of *Proteus mirabilis* isolates to quinolones. Journal of Antimicrobial Chemotherapy, 45(3), 407–408. 10702570 10.1093/jac/45.3.407

[vms31371-bib-0026] Irusen, S. , Rabie, H. , & du Buisson, C. (2021). The evolving antibiotic profile of paediatric urinary tract infections at a tertiary hospital in Cape Town. Wits Journal of Clinical Medicine, 3(1), 25–32.

[vms31371-bib-0027] Ishaq, K. , Ahmad, A. , Rafique, A. , Aslam, R. , Ali, S. , Shahid, M. A. , Sarwar, N. , Aamir, A. M. , Aslam, B. , & Arshad, M. I. (2022). Occurrence and antimicrobial susceptibility of *Proteus mirabilis* from chicken carcass. Pakistan Veterinary Journal, 42(4), 576–579.

[vms31371-bib-0028] Jacoby, G. A. (2009). AmpC β‐lactamases. Clinical Microbiology Reviews, 22(1), 161–182. 19136439 10.1128/CMR.00036-08 PMC2620637

[vms31371-bib-0029] Jamil, B. , Bokhari, H. , & Imran, M. (2017). Mechanism of action: How nano‐antimicrobials act? Current Drug Targets, 18(3), 363–373. 26477460 10.2174/1389450116666151019101826

[vms31371-bib-0030] Karapavlidou, P. , Sofianou, D. , Manolis, E. N. , Pournaras, S. , & Tsakris, A. (2005). CTX‐M‐1 extended‐spectrum β‐lactamase‐producing *Proteus mirabilis* in Greece. Microbial Drug Resistance, 11(4), 351–354. 16359194 10.1089/mdr.2005.11.351

[vms31371-bib-0031] Ko, K. S. , Lee, M. Y. , Song, J. H. , Lee, H. , Jung, D. S. , Jung, S. I. , & Lee, N. Y. (2008). Prevalence and characterization of extended‐spectrum β‐lactamase–producing Enterobacteriaceae isolated in Korean hospitals. Diagnostic Microbiology and Infectious Disease, 61(4), 453–459. 18482815 10.1016/j.diagmicrobio.2008.03.005

[vms31371-bib-0032] Kwiecińska‐Piróg, J. , Skowron, K. , Zniszczol, K. , & Gospodarek, E. (2013). The assessment of *Proteus mirabilis* susceptibility to ceftazidime and ciprofloxacin and the impact of these antibiotics at subinhibitory concentrations on *Proteus mirabilis* biofilms. BioMed Research International, 2013, 930876. 24151628 10.1155/2013/930876 PMC3787586

[vms31371-bib-0033] Lazm, A. M. , Jebur, M. S. , & Alomashi, G. B. (2018). Sequencing of HpmA Gene in *Proteus mirabilis* of UTIs among rheumatoid arthritis patients. Journal of Pharmaceutical Sciences and Research, 10(2), 265–271.

[vms31371-bib-0034] Li, X. , Du, Y. , Du, P. , Dai, H. , Fang, Y. , Li, Z. , Lv, N. , Zhu, B. , Kan, B. , & Wang, D. (2016). SXT/R391 integrative and conjugative elements in *Proteus* species reveal abundant genetic diversity and multidrug resistance. Scientific Reports, 6(1), 1–9. 27892525 10.1038/srep37372 PMC5124997

[vms31371-bib-0035] Li, Z. , Peng, C. , Zhang, G. , Shen, Y. , Zhang, Y. , Liu, C. , & Wang, F. (2022). Prevalence and characteristics of multidrug‐resistant *Proteus mirabilis* from broiler farms in Shandong Province, China. Poultry Science, 1014, 101710. 10.1016/j.psj.2022.101710 PMC8844651 35134599

[vms31371-bib-0036] Lin, M. F. , Liou, M. L. , Kuo, C. H. , Lin, Y. Y. , Chen, J. Y. , & Kuo, H. Y. (2019). Antimicrobial susceptibility and molecular epidemiology of *Proteus mirabilis* isolates from three hospitals in Northern Taiwan. Microbial Drug Resistance, 25(9), 1338–1346. 31295061 10.1089/mdr.2019.0066

[vms31371-bib-0037] Luzzaro, F. , Perilli, M. , Amicosante, G. , Lombardi, G. , Belloni, R. , Zollo, A. , Bianchi, C. , & Toniolo, A. (2001). Properties of multidrug‐resistant, ESBL‐producing *Proteus mirabilis* isolates and possible role of β‐lactam/β‐lactamase inhibitor combinations. International Journal of Antimicrobial Agents, 17(2), 131–135. 11165117 10.1016/s0924-8579(00)00325-3

[vms31371-bib-0038] Lv, F. , Wang, W. , Luo, Y. , Wang, H. , Zhi, T. , Li, X. , Guo, Z. , & Zhao, Z. (2022). Genome‐based analysis of a multidrug‐resistant hypervirulent *Klebsiella pneumoniae* . Microbial Drug Resistance, 28(8), 853–860. 35972766 10.1089/mdr.2021.0307

[vms31371-bib-0039] Manos, J. , & Belas, R. (2006). The genera *Proteus, Providencia*, and *Morganella* . Prokaryotes, 6, 245–269.

[vms31371-bib-0040] Marshall, B. M. , & Levy, S. B. (2011). Food animals and antimicrobials: Impacts on human health. Clinical Microbiology Reviews, 24(4), 718–733. 21976606 10.1128/CMR.00002-11 PMC3194830

[vms31371-bib-0041] Mileng, K. , Ramatla, T. A. , Ndou, R. V. , Thekisoe, O. M. , & Syakalima, M. (2021). Isolation and antibiotic sensitivity of *Campylobacter* species from fecal samples of broiler chickens in North West Province, South Africa. Veterinary World, 14(11), 2929. 35017840 10.14202/vetworld.2021.2929-2935 PMC8743783

[vms31371-bib-0042] Mirzaei, A. , Nasr Esfahani, B. , Raz, A. , Ghanadian, M. , & Moghim, S. (2021). From the urinary catheter to the prevalence of three classes of integrons, β‐lactamase genes, and differences in antimicrobial susceptibility of *Proteus mirabilis* and clonal relatedness with Rep‐PCR. BioMed Research International, 2021, 9952769. 34212042 10.1155/2021/9952769 PMC8211507

[vms31371-bib-0043] Mobley, H. L. , & Belas, R. (1995). Swarming and pathogenicity of *Proteus mirabilis* in the urinary tract. Trends in Microbiology, 3, 280–284. 7551643 10.1016/s0966-842x(00)88945-3

[vms31371-bib-0044] Mokracka, J. , Koczura, R. , & Kaznowski, A. (2012). Multi‐resistant Enterobacteriaceae with class 1 and class 2 integrons in a municipal wastewater treatment plant. Water Research, 46(10), 3353–3363. 22507248 10.1016/j.watres.2012.03.037

[vms31371-bib-0045] Monyama, M. C. , Taioe, O. M. , Nkhebenyane, J. S. , van Wyk, D. , Ramatla, T. , & Thekisoe, O. M. (2023). Bacterial communities associated with houseflies (*Musca domestica* L.) inhabiting hospices in South Africa. Microorganisms, 11(6), 1440. 37374941 10.3390/microorganisms11061440 PMC10304104

[vms31371-bib-0046] Naas, T. , Zerbib, M. , Girlich, D. , & Nordmann, P. (2003). Integration of a transposon Tn 1‐encoded inhibitor‐resistant β‐lactamase gene, bla TEM‐67 from *Proteus mirabilis*, into the *Escherichia coli* chromosome. Antimicrobial Agents and Chemotherapy, 47(1), 19–26. 12499163 10.1128/AAC.47.1.19-26.2003 PMC148959

[vms31371-bib-0047] Nahar, A. , Siddiquee, M. , Nahar, S. , Anwar, K. S. , & Islam, S. (2014). Multidrug resistant‐*Proteus mirabilis* isolated from chicken droppings in commercial poultry farms: Bio‐security concern and emerging public health threat in Bangladesh. Journal of Biosafety & Health Education, 2, 120.

[vms31371-bib-0048] Nana, T. , Bhoora, S. , & Chibabhai, V. (2021). Trends in the epidemiology of urinary tract infections in pregnancy at a tertiary hospital in Johannesburg: Are contemporary treatment recommendations appropriate? Southern African Journal of Infectious Diseases, 36(1), a328. 10.4102/sajid.v36i1.328 PMC8678934 34957292

[vms31371-bib-0049] Ohno, Y. , Nakamura, A. , Hashimoto, E. , Matsutani, H. , Abe, N. , Fukuda, S. , & Nakamura, F. (2017). Molecular epidemiology of carbapenemase‐producing Enterobacteriaceae in a primary care hospital in Japan, 2010–2013. Journal of Infection and Chemotherapy, 23(4), 224–229. 28161293 10.1016/j.jiac.2016.12.013

[vms31371-bib-0050] Pagani, L. , Dell'Amico, E. , Migliavacca, R. , D'Andrea, M. M. , Giacobone, E. , Amicosante, G. , & Rossolini, G. M. (2003). Multiple CTX‐M‐type extended‐spectrum β‐lactamases in nosocomial isolates of Enterobacteriaceae from a hospital in northern Italy. Journal of Clinical Microbiology, 41(9), 4264–4269. 12958255 10.1128/JCM.41.9.4264-4269.2003 PMC193787

[vms31371-bib-0051] Pagani, L. , Migliavacca, R. , Pallecchi, L. , Matti, C. , Giacobone, E. , Amicosante, G. , & Rossolini, G. M. (2002). Emerging extended‐spectrum β‐lactamases in *Proteus mirabilis* . Journal of Clinical Microbiology, 40(4), 1549–1552. 11923394 10.1128/JCM.40.4.1549-1552.2002 PMC140357

[vms31371-bib-0052] Paterson, D. L. (2006). Resistance in gram‐negative bacteria: Enterobacteriaceae. American Journal of Infection Control, 34(5), S20–S28. 16813978 10.1016/j.ajic.2006.05.238

[vms31371-bib-0053] Pitout, J. D. D. , Thomson, K. S. , Hanson, N. D. , Ehrhardt, A. F. , Moland, E. S. , & Sanders, C. C. (1998). β‐Lactamases responsible for resistance to expanded‐spectrum cephalosporins in *Klebsiella pneumoniae*, *Escherichia coli*, and *Proteus mirabilis* isolates recovered in South Africa. Antimicrobial Agents and Chemotherapy, 42(6), 1350–1354. 9624474 10.1128/aac.42.6.1350 PMC105602

[vms31371-bib-0054] Pitout, J. D. , Nordmann, P. , Laupland, K. B. , & Poirel, L. (2005). Emergence of Enterobacteriaceae producing extended‐spectrum β‐lactamases (ESBLs) in the community. Journal of Antimicrobial Chemotherapy, 56(1), 52–59. 15917288 10.1093/jac/dki166

[vms31371-bib-0055] Racewicz, P. , Majewski, M. , Biesiada, H. , Nowaczewski, S. , Wilczyński, J. , Wystalska, D. , Kubiak, M. , Pszczoła, M. , & Madeja, Z. E. (2022). Prevalence and characterisation of antimicrobial resistance genes and class 1 and 2 integrons in multiresistant *Escherichia coli* isolated from poultry production. Scientific Reports, 12(1), 1–13. 35410349 10.1038/s41598-022-09996-y PMC9001716

[vms31371-bib-0056] Rajivgandhi, G. , Maruthupandy, M. , & Manoharan, N. (2018). Detection of TEM and CTX‐M genes from ciprofloxacin resistant *Proteus mirabilis* and *Escherichia coli* isolated on urinary tract infections (UTIs). Microbial Pathogenesis, 121, 123–130. 29778819 10.1016/j.micpath.2018.05.024

[vms31371-bib-0057] Ramatla, T. , Mileng, K. , Ndou, R. , Mphuti, N. , Syakalima, M. , Lekota, K. E. , & Thekisoe, O. M. (2022). Molecular detection of integrons, colistin and β‐lactamase resistant genes in *Salmonella enterica* serovars enteritidis and typhimurium isolated from chickens and rats inhabiting poultry farms. Microorganisms, 10(2), 313. 35208768 10.3390/microorganisms10020313 PMC8876313

[vms31371-bib-0058] Ramatla, T. , Taioe, M. O. , Thekisoe, O. M. , & Syakalima, M. (2019). Confirmation of antimicrobial resistance by using resistance genes of isolated *Salmonella* spp. in chicken houses of North West, South Africa. World's Veterinary Journal, 9(3), 158–165.

[vms31371-bib-0059] Ramatla, T. , Tutubala, M. , Motlhaping, T. , de Wet, L. , Mokgokong, P. , Thekisoe, O. , & Lekota, K. (2024). Molecular detection of Shiga toxin and extended‐spectrum beta‐lactamase (ESBL)‐producing *Escherichia coli* isolates from sheep and goats. Molecular Biology Reports, 51(1), 57. 38165462 10.1007/s11033-023-08987-0 PMC10761393

[vms31371-bib-0060] Rao, S. P. , Rama, P. S. , Gurushanthappa, V. , Manipura, R. , & Srinivasan, K. (2014). Extended‐spectrum beta‐lactamases producing *Escherichia coli* and *Klebsiella pneumoniae*: A multi‐centric study across Karnataka. Journal of Laboratory Physicians, 6(01), 007–013. 10.4103/0974-2727.129083 PMC3969652 24696553

[vms31371-bib-0061] Sanches, M. S. , Baptista, A. A. S. , de Souza, M. , Menck‐Costa, M. F. , Justino, L. , Nishio, E. K. , & Rocha, S. P. D. (2020). *Proteus mirabilis* causing cellulitis in broiler chickens. Brazilian Journal of Microbiology, 51, 1353–1362. 32067208 10.1007/s42770-020-00240-1 PMC7455630

[vms31371-bib-0062] Sanches, M. S. , Baptista, A. A. S. , de Souza, M. , Menck‐Costa, M. F. , Koga, V. L. , Kobayashi, R. K. T. , & Rocha, S. P. D. (2019). Genotypic and phenotypic profiles of virulence factors and antimicrobial resistance of *Proteus mirabilis* isolated from chicken carcasses: Potential zoonotic risk. Brazilian Journal of Microbiology, 50, 685–694. 31049879 10.1007/s42770-019-00086-2 PMC6863274

[vms31371-bib-0063] Sanches, M. S. , Silva, L. C. , Silva, C. R. D. , Montini, V. H. , Oliva, B. H. D. D. , Guidone, G. H. M. , Nogueira, M. C. L. , Menck‐Costa, M. F. , Kobayashi, R. K. T. , Vespero, E. C. , & Rocha, S. P. D. (2023). Prevalence of antimicrobial resistance and clonal relationship in ESBL/AmpC‐producing *Proteus mirabilis* isolated from meat products and community‐acquired urinary tract infection (UTI‐CA) in Southern Brazil. Antibiotics, 12(2), 370. 36830280 10.3390/antibiotics12020370 PMC9952622

[vms31371-bib-0064] Santos, M. M. D. , Alcântara, A. C. M. D. , Perecmanis, S. , Campos, A. , & Santana, A. P. (2014). Antimicrobial resistance of bacterial strains isolated from avian cellulitis. Brazilian Journal of Poultry Science, 16, 13–18.

[vms31371-bib-0065] Seo, K. W. , & Lee, Y. J. (2019). Prevalence and characterization of plasmid mediated quinolone resistance genes and class 1 integrons among multidrug‐resistant *Escherichia coli* isolates from chicken meat. Journal of Applied Poultry Research, 28(3), 761–770.

[vms31371-bib-0066] Shaaban, M. , Elshaer, S. L. , El‐Rahman, A. , & Ola, A. (2022). Prevalence of extended‐spectrum β‐lactamases, *AmpC*, and carbapenemases in *Proteus mirabilis* clinical isolates. BMC Microbiology, 22(1), 1–13. 36221063 10.1186/s12866-022-02662-3 PMC9552493

[vms31371-bib-0067] Sherchan, J. B. , Hayakawa, K. , Miyoshi‐Akiyama, T. , Ohmagari, N. , Kirikae, T. , Nagamatsu, M. , Tojo, M. , Ohara, H. , Sherchand, J. B. , & Tandukar, S. (2015). Clinical epidemiology and molecular analysis of extended‐spectrum‐β‐lactamase‐producing *Escherichia coli* in Nepal: Characteristics of sequence types 131 and 648. Antimicrobial Agents and Chemotherapy, 59(6), 3424–3432. 25824221 10.1128/AAC.00270-15 PMC4432170

[vms31371-bib-0068] Somily, A. M. , Arshad, M. Z. , Garaween, G. A. , & Senok, A. C. (2015). Phenotypic and genotypic characterization of extended‐spectrum b‐lactamases producing *Escherichia coli* and *Klebsiella pneumoniae* a tertiary care hospital in Riyadh, Saudi Arabia. Annals of Saudi Medicine, 35(6), 435–439. 26657226 10.5144/0256-4947.2015.435 PMC6074471

[vms31371-bib-0070] Song, W. , Kim, J. , Bae, I. K. , Jeong, S. H. , Seo, Y. H. , Shin, J. H. , & Lee, K. (2011). Chromosome‐encoded AmpC and CTX‐M extended‐spectrum β‐lactamases in clinical isolates of *Proteus mirabilis* from Korea. Antimicrobial Agents and Chemotherapy, 55(4), 414–1419. 21282448 10.1128/AAC.01835-09 PMC3067170

[vms31371-bib-0071] Sun, Y. , Wen, S. , Zhao, L. , Xia, Q. , Pan, Y. , Liu, H. , Wei, C. , Chen, H. , Ge, J. , & Wang, H. (2020). Association among biofilm formation, virulence gene expression, and antibiotic resistance in *Proteus mirabilis* isolates from diarrhetic animals in Northeast China. BMC Veterinary Research, 16(1), 1–10. 32503535 10.1186/s12917-020-02372-w PMC7275385

[vms31371-bib-0072] Tewari, R. , Mitra, S. , Venugopal, N. , Das, S. , Ganaie, F. , Sen, A. , & Shome, B. R. (2019). Phenotypic and molecular characterization of extended spectrum β‐lactamase, *ampc* β‐lactamase and metallo β‐lactamase producing *Klebsiella* spp. from farm animals in India. Indian Journal of Animal Research, 53(7), 938–943.

[vms31371-bib-0073] Tsai, H. Y. , Chen, Y. H. , Tang, H. J. , Huang, C. C. , Liao, C. H. , Chu, F. Y. , & Hsueh, P. R. (2014). Carbapenems and piperacillin/tazobactam for the treatment of bacteremia caused by extended‐spectrum *β*‐lactamase–producing *Proteus mirabilis* . Diagnostic Microbiology and Infectious Disease, 80(3), 222–226. 25139843 10.1016/j.diagmicrobio.2014.07.006

[vms31371-bib-0074] Uphoff, T. S. , & Welch, R. A. (1990). Nucleotide sequencing of the Proteus mirabilis calcium‐independent hemolysin genes (*hpmA* and *hpmB*) reveals sequence similarity with the *Serratia marcescens* hemolysin genes (*shlA* and *shlB*). Journal of Bacteriology, 172(3), 1206–1216. 2407716 10.1128/jb.172.3.1206-1216.1990 PMC208585

[vms31371-bib-0075] Ushie, S. N. , Oyedeji, K. S. , Ogban, G. I. , Ushie, D. E. , Nwaokorie, F. O. , Odeniyi, O. M. , & Ezeador, C. O. (2020). Molecular epidemiology of extended spectrum beta‐lactamases producing *Escherichia coli* and *Klebsiella* species in catheterized patients. European Journal of Medical and Health Sciences, 2(4), 2020.

[vms31371-bib-0076] von Tippelskirch, P. , Gölz, G. , Projahn, M. , Daehre, K. , Friese, A. , Roesler, U. , Alter, T. , & Orquera, S. (2018). Prevalence and quantitative analysis of ESBL and AmpC beta‐lactamase producing Enterobacteriaceae in broiler chicken during slaughter in Germany. International Journal of Food Microbiology, 281, 82–89. 29890401 10.1016/j.ijfoodmicro.2018.05.022

[vms31371-bib-0077] Wang, J. , Zhu, X. , Zhao, Y. , Liu, H. , Zhang, Z. , Yan, L. , & Aleri, J. W. (2023). Prevalence and antimicrobial resistance of *Salmonella* and ESBL *E. coli* isolated from dairy cattle in Henan Province, China. Preventive Veterinary Medicine, 213, 105856. 36716653 10.1016/j.prevetmed.2023.105856

[vms31371-bib-0078] Warren, J. W. , Tenney, J. H. , Hoopes, J. M. , Muncie, H. L. , & Anthony, W. C. (1982). A prospective microbiologic study of bacteriuria in patients with chronic indwelling urethral catheters. Journal of Infectious Diseases, 146, 719–723. 6815281 10.1093/infdis/146.6.719

[vms31371-bib-0079] Wasfi, R. , Hamed, S. M. , Amer, M. A. , & Fahmy, L. I. (2020). *Proteus mirabilis* biofilm: Development and therapeutic strategies. Frontiers in Cellular and Infection Microbiology, 10, 414. 32923408 10.3389/fcimb.2020.00414 PMC7456845

[vms31371-bib-0080] Wu, D. , Ding, Y. , Yao, K. , Gao, W. , & Wang, Y. (2021). Antimicrobial resistance analysis of clinical *Escherichia coli* isolates in neonatal ward. Frontiers in Pediatrics, 9, 670470. 34113589 10.3389/fped.2021.670470 PMC8185016

[vms31371-bib-0081] Yarima, A. , Haroun, A. A. , Bulus, T. , & Manga, M. M. (2020). Occurrence of extended spectrum beta lactamase encoding genes among urinary pathogenic *Escherichia coli* and *Klebsiella pneumoniae* isolates obtained from a Tertiary Hospital in Gombe Nigeria. Journal of Biosciences and Medicines, 8(09), 42.

