# Supplementary material for: Antibiotic resistance and virulence profiles of Proteus mirabilis isolated from broiler chickens at abattoir in South Africa
Source: Vet Med Sci. 2024 Feb 15;10(2):e1371. doi: 10.1002/vms3.1371 (PMC10867704; doi:10.1002/vms3.1371)
Supplement: Supplementary file 1 — SUPPORTING INFORMATION [file VMS3-10-e1371-s001.docx]

**Antibiotic resistance and virulence profiles of *Proteus mirabilis* isolated from broiler chickens at abattoir in South Africa**

**Table S1.** Antibiotic resistance genes, PCR primers and conditions.

| **Primer** |  | **Sequence of primers (5–3’)** | **Size (bp)** | **Annealing Temp (°C)** | **Reference** |
| --- | --- | --- | --- | --- | --- |
| *qnrS* | FW | CCCCATGCCCGAAGTTATCA | 457 | 53 | Xu et al., 2015 |
|  | RV | ACTGCTTGGAGTGTGTTGGT |  |  |  |
| *qnrA* | FW | ATTTCTCACGCCAGGATTTG | 413 | 53 | Huang et al., 2012 |
|  | RV | GAGATTGGCATTGCTCCAGT |  |  |  |
| *qnrD* | FW | GCTGGAGCTTGTCAGGGATT | 585 | 59 | Xu et al., 2015 |
|  | RV | TGCTGCGAGATATCATGCGT |  |  |  |
| *catI* | FW | GGTGATATGGGATAGTGTT | 349 | 60 | Jiang et al., 2013 |
|  | RV | CCATCACATACTGCATGATG |  |  |  |
| *catII* | FW | GATTGACCTGAATACCTGGAA | 567 | 60 | Jiang et al., 2013 |
|  | RV | CCATCACATACTGCATGATG |  |  |  |
| *vanA* | FW | GAAATCAACCATGTTGATGTAGCA | 572 | 60 | Böckelmann et al., 2009 |
|  | RV | TTTGCCGTTTCCTGTATCCGT |  |  |  |
| *ermB* | FW | GCATTTAACGACGAAACTGGCT | 573 | 60 | Malik et al., 2008 |
|  | RV | GACAATACTTGCTCATAAGTAATGGT |  |  |  |
| *mecA* | FW | CAAGTACTTTYAAAACAATAGC | 534 | 61 | Jiang et al., 2013 |
|  | RV | GCTGTAATACTCCKAGCAC |  |  |  |
|  | RV | AAAATACTGCGTGGCAGGTAGC |  |  |  |
| *int1* | FW | GCCTTGCTGTTCTTCTACGG | 558 | 65 | Sedighi et al., 2017 |
|  | RV | GATGCCTGCTTGTTCTACGG |  |  |  |
| *int2* | FW | CAC GGA TAT GCGACA AAA AGG | 789 | 65 | Sedighi et al., 2017 |
|  | RV | TGTA GCA AAC GAGTGA CGA AAT G |  |  |  |
| *amp*C | FW | GTGACCAGATACTGGCCACA | 822 | 61 | Jiang et al., 2013 |
|  | RV | TTACTGTAGCGCCTCGAGGA |  |  |  |
| *SHV* |  | CACTCAAGGATGTATTGT G | 885 | 55 | Ramatla et al., 2022 |
|  |  | TTAGCGTTGCCAGTGCTCG |  |  |  |
| *OXA* | FW | ACACAATACATATCAACTTCGC | 813 | 55 | Ramatla et al., 2022 |
|  | RV | AGTGTGTTTAGAATGGTGATC |  |  |  |
| *CARB* | FW | CAAGTACTTTYAAAACAATAGC | 534 | 46 | Jiang et al., 2013 |
|  | RV | GCTGTAATACTCCKAGCAC |  |  |  |
| *TEM* | FW | TTC TTG AAG ACG AAA GGG C | 1150 | 55 | Ramatla et al., 2022 |
|  | RV | ACGCTCAGTGGAACGAAAAC |  |  |  |
| *CTX-M* | FW | GTTACAATGTGTGAGAAGCAG | 550 | 55 | Liu et al., 2018 |
|  | RV | CCGTTTCCGCTATTACAAAC |  |  |  |
| *CTX-M-1* group | FW | GTTACA ATG TGT GAG AAG CAG | 1041 | 55 | Liu et al., 2018 |
|  | RV | CCGTTTCCGCTATTACAAAC |  |  |  |
| *CTX-M-2* group | FW | ATGATGACTCAGAGCATTCGCCGC | 876 | 55 | Liu et al., 2018 |
|  | RV | TCAGAAACCGTGGGTTACGATTTT |  |  |  |
| *CTX-M-8* group | FW | TGATGAGACATCGCGTTAAG | 666 | 55 | Gundran et al., 2019 |
|  | RV | TAACCGTCGGTGACGATTTT |  |  |  |
| *CTX-M-9* group | FW | GTGACAAAGAGAGTGCAACGG | 856 | 55 | Gundran et al., 2019 |
|  | RV | ATGATTCTCGCCGCTGAAGCC |  |  |  |
| *CTX-M-15* group | FW | CACACGTGGAATTTAGGGACT | 995 | 55 | Liu et al., 2018 |
|  | RV | GCCGTCTAAGGCGATAAACA |  |  |  |
| *CTX-M-25* group | FW | GCACGATGACATTCGGG | 327 | 55 | Gundran et al., 2019 |
|  | RV |  |  |  |  |

**Table S2.** PCR primers of virulence genes screened from *P. mirabilis* isolates.

| **Genes** | **Primers sequences** | **PCR conditions and cycles** | **Base pairs** | **References** |
| --- | --- | --- | --- | --- |
| *pmfA* | F- CAAATTAATCTAGAACCACTC | 1 cycle of 5 minutes at 95°C, 35 cycles of 1 minutes at 95°C, 1 minute at 54°C, 1 minute at 72°C; 1 cycle of 7 minutes at 72°C. | 617 | Sanches et al., 2019 |
|  | R- ATTATAGAGGATCCCTTGAAGGTA |  |  |  |
| *ireA* | F- ACTACGATAACGAGCGCCAG | 1 cycle of 5 minutes at 95°C, 35 cycles of 1 minutes at 95°C, 1 minute at 60°C, 1 minute at 72°C; 1 cycle of 7 minutes at 72°C. | 681 | Sanches et al., 2019 |
|  | R- GCCCTAACTGGGGGAATACG |  |  |  |
| *ucaA* | F- GCTTTTACATCCCCAGCGGT | 1 cycle of 5 minutes at 95°C, 35 cycles of 1 minutes at 95°C, 1 minute at 60°C, 1 minute at 72°C; 1 cycle of 7 minutes at 72°C. | 476 | Sanches et al., 2019 |
|  | R- GCTGCATTTGCTGGCTCATC |  |  |  |
| *ptA* | F- CCACTGCGATTATCCGCTCT | 1 cycle of 5 minutes at 95°C, 35 cycles of 1 minutes at 95°C, 1 minute at 60°C, 1 minute at 72°C; 1 cycle of 7 minutes at 72°C. | 686 | Sanches et al., 2019 |
|  | R- ATCGGCAGAAGTGACAAGCA |  |  |  |
| *zapA* | F- TATCGTCTCCTTCGCCTCCA | 1 cycle of 5 minutes at 95°C, 35 cycles of 1 minutes at 95°C, 1 minute at 59°C, 1 minute at 72°C; 1 cycle of 7 minutes at 72°C. | 332 | Sanches et al., 2019 |
|  | R- TGGCGCAAATACGACTACCA |  |  |  |
| *hpmA* | F- GTTGAGGGGCGTTATCAAGAGTC | 1 cycle of 5 minutes at 95°C, 35 cycles of 1 minutes at 95°C, 1 minute at 55°C, 1 minute at 72°C; 1 cycle of 7 minutes at 72°C. | 709 | Cestari et al., 2013 |
|  | R- GATACTGTTTTGCCCTTTTGTGC |  |  |  |
| *hlyA* | F- AACAAGGATAAGCACTGTTCTGGCT | 1 cycle of 5 minutes at 95°C, 35 cycles of 1 minutes at 95°C, 1 minute at 563°C, 1 minute at 72°C; 1 cycle of 7 minutes at 72°C. | 1177 | Cestari et al., 2013 |
|  | R- ACCATATAAGCGGTCATTCCCGTCA |  |  |  |
| *mrpA* | F- GAGCCATTCAATTAGGAATAATCCA | 1 cycle of 5 minutes at 95°C, 35 cycles of 1 minutes at 95°C, 1 minute at 58°C, 1 minute at 72°C; 1 cycle of 7 minutes at 72°C. | 648 | Rocha et al., 2007 |
|  | R- AGCTCTGTACTTCCTTGTACAGA |  |  |  |
| *atfA* | F- CATAATTTCTAGACCTGCCCTAGCA | 1 cycle of 5 minutes at 95°C, 35 cycles of 1 minutes at 95°C, 1 minute at 50°C, 1 minute at 72°C; 1 cycle of 7 minutes at 72°C. | 382 | Zunino et al., 2000 |
|  | R- CTGCTTGGATCCGTAATTTTTAACG |  |  |  |

**References**

1. Sanches, M.S., Baptista, A.A.S., de Souza, M., Menck-Costa, M.F., Koga, V.L., Kobayashi, R.K.T. and Rocha, S.P.D., 2019. Genotypic and phenotypic profiles of virulence factors and antimicrobial resistance of *Proteus mirabilis* isolated from chicken carcasses: potential zoonotic risk. Brazilian Journal of Microbiology, 50(3), pp.685-694.
2. Cestari, S.E., Ludovico, M.S., Martins, F.H., da Rocha, S.P.D., Elias, W.P. and Pelayo, J.S., 2013. Molecular detection of HpmA and HlyA hemolysin of uropathogenic *Proteus mirabilis*. *Current microbiology*, 67(6), pp.703-707.
3. Rocha, S.P., Elias, W.P., Cianciarullo, A.M., Menezes, M.A., Nara, J.M., Piazza, R.M., Silva, M.R., Moreira, C.G. and Pelayo, J.S., 2007. Aggregative adherence of uropathogenic Proteus mirabilis to cultured epithelial cells. FEMS Immunology & Medical Microbiology, 51(2), pp.319-326.\
4. Zunino, P., Geymonat, L., Allen, A.G., Legnani-Fajardo, C. and Maskell, D.J., 2000. Virulence of a *Proteus mirabilis* ATF isogenic mutant is not impaired in a mouse model of ascending urinary tract infection. FEMS Immunology & Medical Microbiology, 29(2), pp.137-143.
5. Xu, Y., Yu, W., Ma, Q. and Zhou, H., 2015. Occurrence of (fluoro) quinolones and (fluoro) quinolone resistance in soil receiving swine manure for 11 years. Science of the Total Environment, 530, pp.191-197.
6. Huang S, Dai W, Sun S, Zhang X, Zhang L (2012) Prevalence of Plasmid-Mediated Quinolone Resistance and Aminoglycoside Resistance Determinants among Carbapeneme Non-Susceptible Enterobacter cloacae. PLoS ONE 7(10): e47636. <https://doi.org/10.1371/journal.pone.0047636>
7. Böckelmann, U., Dörries, H.H., Ayuso-Gabella, M.N., Salgot de Marçay, M., Tandoi, V., Levantesi, C., Masciopinto, C., Van Houtte, E., Szewzyk, U., Wintgens, T. and Grohmann, E., 2009. Quantitative PCR monitoring of antibiotic resistance genes and bacterial pathogens in three European artificial groundwater recharge systems. Applied and environmental microbiology, 75(1), pp.154-163.
8. Malik, A., Çelik, E.K., Bohn, C., Böckelmann, U., Knobel, K. and Grohmann, E., 2008. Detection of conjugative plasmids and antibiotic resistance genes in anthropogenic soils from Germany and India. FEMS microbiology letters, 279(2), pp.207-216.
9. Sedighi, M., Halajzadeh, M., Ramazanzadeh, R., Amirmozafari, N., Heidary, M. and Pirouzi, S., 2017. Molecular detection of β-lactamase and integron genes in clinical strains of *Klebsiella pneumoniae* by multiplex polymerase chain reaction. Revista da Sociedade Brasileira de Medicina Tropical, 50, pp.321-328.
10. Jiang, L., Hu, X., Xu, T., Zhang, H., Sheng, D. and Yin, D., 2013. Prevalence of antibiotic resistance genes and their relationship with antibiotics in the Huangpu River and the drinking water sources, Shanghai, China. *Science of the Total Environment*, *458*, pp.267-272.
